# Addressing the Opioid Crisis—The Need for a Pain Management Intervention in Community Pharmacies in Canada: A Narrative Review

**DOI:** 10.3390/pharmacy11020071

**Published:** 2023-04-06

**Authors:** Ashley Cid, Angeline Ng, Victoria Ip

**Affiliations:** 1 Ontario Pharmacists’ Association, Toronto, ON M5H 3B7, Canada; 2 School of Pharmacy, University of Waterloo, Kitchener, ON N2G 1C5, Canada

**Keywords:** opioid crisis, pain management, community pharmacy, prescription opioids

## Abstract

**Background:** The opioid crisis is a public health concern in Canada with a continued rise in deaths and presents a significant economic impact on the healthcare system. There is a need to develop and implement strategies for decreasing the risk of opioid overdoses and other opioid-related harms resulting from the use of prescription opioids. Pharmacists, as medication experts and educators, and as one of the most accessible frontline healthcare providers, are well positioned to provide effective opioid stewardship through a pain management program focused on improving pain management for patients, supporting appropriate prescribing and dispensing of opioids, and supporting safe and appropriate use of opioids to minimize potential opioid misuse, abuse, and harm. **Methods:** A literature search was conducted in PubMed, Embase and grey literature to determine the characteristics of an effective community pharmacy-based pain management program, including the facilitators and barriers to be considered. **Discussion:** An effective pain management program should be multicomponent, address other co-morbid conditions in addition to pain, and contain a continuing education component for pharmacists. Solutions to implementation barriers, including pharmacy workflow; addressing attitudes beliefs, and stigma; and pharmacy remuneration, as well as leveraging the expansion of scope from the *Controlled Drugs and Substances Act* exemption to facilitate implementation, should be considered. **Conclusions:** Future work should include the development, implementation, and evaluation of a multicomponent, evidence-based intervention strategy in Canadian community pharmacies to demonstrate the impact pharmacists can have on the management of chronic pain and as one potential solution to helping curb the opioid crisis. Future studies should measure associated costs for such a program and any resulting cost-savings to the healthcare system.

## 1. Introduction

### 1.1. Overview of the Opioid Crisis

The opioid crisis continues to worsen every year in Canada and is a major public health concern [1]. Between January 2016 and March 2022, there were a total of 30,843 apparent opioid toxicity deaths and 32,319 opioid-related poisoning hospitalizations in Canada [2,3]. The COVID-19 pandemic played a significant part in contributing to opioid-related harms and deaths, as the first two years of the pandemic (April 2020 to March 2022) were associated with a 91% increase in apparent opioid toxicity deaths and a 24% increase in opioid-related poisoning hospitalizations nationally compared to the two years prior (April 2018 to March 2020) [2,3].

Although illicit opioids are a major contributor to the opioid crisis, there is evidence that a surge in opioid prescriptions may also be a key factor [4]. Pain is one of the most common reasons for accessing healthcare in North America, and one commonly used management approach is prescription opioids [5]. In 2018, one in eight Canadians were prescribed opioids [5]. Long-term use of opioids can lead to physical dependence and/or tolerance, which results in higher doses being used to achieve the desired effects [5]. The repeated use of higher doses increases the potential for addiction [5]. In some situations, prescription opioids can lead to opioid misuse and an increased risk of progression to opioid use disorder (OUD) [4]. Individuals with OUD may access prescription opioids through various means including, but not limited to from one physician, from multiple physicians without informing them of other prescriptions (double doctoring), prescription fraud or forgery, theft, street drug markets, and Internet purchases [5]. In 2017, approximately 37% of Canadians with OUD reported accessing opioids solely through regulated (legal) channels (i.e., prescriptions), and a further 26% reported obtaining their opioids through both prescription and unregulated (illegal) channels [4]. Therefore, it is critical that the development and implementation of strategies to address the opioid crisis not only focus on illicit opioids but also the role that prescribed opioids can have on the opioid epidemic.

Strategies to manage the opioid crisis not only improve patient health outcomes but can also have a significant economic impact. In 2017, the economic cost of opioid substance use in Canada was CAD $5.9 billion, or approximately CAD $163 for every Canadian regardless of age (a 20.9% increase from $135 in 2015) [6]. Specifically, with respect to healthcare costs, opioid substance use cost the healthcare system CAD $439 million in 2017 [6]. As the opioid crisis continues to worsen each year, it is likely that the costs associated with opioid substance use will also increase correspondingly. Hence, the development of alternative strategies to manage opioid use can lead to significant potential cost savings for the healthcare system.

### 1.2. Role of Pharmacists as Opioid Stewards

Pharmacists, being one of the most accessible frontline healthcare providers, are well positioned to provide and advocate for effective opioid stewardship, which is described by the Institute for Safe Medication Practices (ISMP) Canada as “coordinated interventions designed to improve, monitor, and evaluate the use of opioids in order to support and protect human health” [7]. Over 91% of Ontarians live within 5 km of a pharmacy and many community pharmacies are open extended hours and, in some instances open 24 h, which increase patient accessibility and convenience [4,8]. Access to community pharmacies is especially important for those living in rural areas who may have limited access to other healthcare services [8]. With respect to managing chronic pain, access to specialist pain treatment services is limited in many regions in Canada resulting in about 50% of patients having to wait over 6 months for appropriate treatment [5]. As opioids are commonly prescribed to manage chronic pain, pharmacists have a key role to play in ensuring their appropriate use [5]. Evidence has shown that taking an interdisciplinary approach, inclusive of pharmacists, to recognize and address the clinical factors that play a role in unnecessary or excessive prescribing and dosing of opioids can enhance clinical outcomes [4].

Pharmacists are medication experts and educators. They can review all available therapeutic options with a patient and provide patient-centered care by recommending evidence-based options to treat chronic or acute pain [9]. They work with prescribers to manage medication dosing, including titrations, cross tapers, and deprescribing/discontinuation of opioids [9]. Pharmacists also possess the knowledge to assess medication regimens to identify and address issues such as drug interactions (pharmacokinetic and pharmacodynamic) and polypharmacy [9]. In applying their knowledge and expertise, community pharmacists are often the first to identify an issue with an opioid prescription [10]. A survey of pharmacists found that of every 10 opioid prescriptions, 1–3 were associated with concerns, such as long-acting opioids being prescribed instead of short-acting (54%), long-acting opioids being prescribed to opioid-naïve patients (38%), patients with mild-to-moderate pain being prescribed opioids rather than being managed with non-opioid options (63%), patients intolerant to opioids and at increased risk for adverse effects being prescribed opioids (63%), and drug interactions (56%) [11]. This highlights the variety of issues pharmacists navigate when presented with an opioid prescription and showcases the important role they play as opioid stewards and the final gatekeeper before the prescription reaches the patient. After ensuring the therapeutic appropriateness of the medication for a patient, pharmacists act as an educator to ensure their patient and/or the patient’s agent is familiar with what to expect when taking an opioid, potential side effects, how to monitor progress, and ongoing management [9]. Patients see their pharmacist 10 times more often than their primary care provider for the management of chronic conditions, including for pain [9]. Therefore, pharmacists are ideally positioned to help patients manage chronic pain, such as providing more frequent follow-up; monitoring; promoting harm reduction by offering naloxone kits, clean needles, and sharps containers; and providing education about how to reduce the risk of an opioid overdose, the safe storage of opioids, and the safe disposal and destruction of unused opioids to safeguard against diversion [9]. A study found that in comparison to prescribers, pharmacists were more likely to implement and document risk-reduction strategies and co-prescribe naloxone for high-risk patients on opioid therapy [12]. This not only demonstrates the important role that pharmacists play in ensuring patients receive timely and appropriate care, but also illustrates the opportunity to enable greater capacity in the health system by better engaging pharmacists as opioid stewards to provide pain management interventions, and collaborating with primary care providers to support their patients.

Pharmacist-led interventions have been shown to have a positive impact on addressing the opioid crisis. One study showed that when pharmacists integrated an opioid misuse risk screening tool into their practice, they identified that 26% of patients prescribed opioids were at some risk of misuse and 30% were at risk for an accidental overdose [13]. The identification of at-risk patients enables pharmacists to provide more targeted counselling on the use of prescribed opioids to patients who may need it most. Furthermore, studies have demonstrated that pharmacist-led interventions are successful in promoting safe and effective prescribing and use of opioids for chronic non-cancer pain [12,14]. A study on a pharmacist-led telephone risk assessment clinic saw the pharmacist recommending changes to chronic opioid prescriptions for one-third of the assessed patients, including decreasing the dispensed quantity, discontinuing the medication, or delaying the refill [12]. Research has also shown that pharmacist-led medication reviews can result in significant decreases to mean morphine equivalent (MME) doses for patients with chronic non-cancer pain [15]. This is important as there is a correlation between the MME and opioid-related overdose deaths [15].

The role pharmacists can play in addressing the opioid crisis is valuable and should be better leveraged in the community setting. However, currently in Canada, there is no standard intervention or remuneration framework for opioid stewardship in community pharmacies. The objective of this narrative review is to describe the required characteristics of an effective pharmacy-based pain management intervention with consideration for its facilitators and barriers. This review is meant to guide the development of a future community pharmacist-led intervention in Ontario, Canada that specifically works to (1) improve pain management for patients, (2) support appropriate prescribing and dispensing of opioids, and (3) support safe and appropriate use of opioids.

## 2. Methods

For this narrative review, our intent was to provide an overview of published literature and highlight select studies showcasing what should be considered when building an effective pharmacy-based pain management intervention, including specific barriers and facilitators that would need to be considered. A literature search was conducted in the PubMed, Embase and Google Scholar databases. These databases were used because they are widely indexed and contain pharmacy and healthcare professional specific literature. Other databases like Web of Science or Scopus were not searched, as they index MEDLINE, which is already included in searches with PubMed and Embase [16]. Grey literature was searched to determine if other countries, jurisdictions, organizations, or pharmacy professional associations have reported unpublished findings related to pharmacist-led pain management interventions.

The following search terms were used in the PubMed, Embase and Google Scholar databases: “pharmacists,” “community pharmacy”, “pharmacist service”, “intervention”, “pain management”, “chronic pain/drug therapy”, “pain clinic”, “pain program”, “program evaluation”, “professional role”, “barrier”, and “facilitator”. Citations from included articles, and similar articles recommended in the PubMed, Embase and Google Scholar databases were also evaluated for inclusion. In addition to searching Google Scholar, a search for relevant grey literature was done using the Google search engine, using the same keywords listed above. Articles were included if they were describing a community pharmacist-led opioid or pain management intervention, or a description of specific factors such as barriers and or facilitators for pharmacist implementation of opioid or pain management interventions. Articles were excluded if they did not include community pharmacists or were interventions not led by pharmacists. Articles before 2013 were excluded as they may not be as relevant to the developing opioid crisis. There was no exclusion based on article or study type; however, only articles in English were considered. Potential articles were independently reviewed by each author for inclusion based on the inclusion and exclusion criteria.

## 3. Results

A review of the literature for studies on pharmacist interventions that could mitigate the opioid crisis by reducing the risk of potential opioid misuse, diversion, and opioid-related harm, revealed certain characteristics that should be incorporated into any pharmacist pain management program to support greater success. A total of 16 articles were selected for inclusion. Descriptions of the included studies are presented below, with an overview of pharmacist-led interventions located in Table A1, and the factors to consider when implementing an intervention in Table A2. Discussion of the articles are presented by theme: multicomponent interventions; management of other comorbidities; continuing education; pharmacy workflow; attitudes, beliefs, and stigma; renumeration; and expanded scope.

### 3.1. Factors to Consider When Implementing an Intervention

Multicomponent Interventions—The literature review identified several studies describing multicomponent interventions as more effective for managing chronic pain when compared to offering only patient education or standard counselling [15,17,18]. Cochran et al. conducted a small randomized controlled study involving 32 patients using an intervention called the Brief Motivational Intervention-Medication Therapy Management (BMI-MTM) strategy combined with standard medication counselling (SMC) compared to only SMC for patients identified as misusing their prescribed opioid medication [17]. They found that BMI-MTM was a feasible misuse intervention and was linked to superior satisfaction and outcomes [17]. BMI-MTM is comprised of four evidence-based practices: medication therapy management (MTM), brief motivational interviewing (BMI), patient navigation, and naloxone training and referral [17]. Pharmacists in the study provided the MTM component in conjunction with BMI with a goal of improving patient adherence to taking the opioid medication as prescribed [17]. It involved a review of the patient’s opioid medications and identifying interactions, if any; a discussion about misuse and any identified misuse behaviours; and the identification of adherence improvement targets as well as providing the patient encouragement to work towards behaviour change [17]. The patient navigation component was conducted by a patient navigator who was a Master’s level research interventionist and was comprised of eight telephone sessions dedicated to first establishing a therapeutic alliance/rapport with the patient; setting goals for needed services and identifying barriers and solutions; aiding patients with enrolling in psychosocial services, behavioural health, and/or physical healthcare; discussing overdose risk and training/referral to obtain a naloxone kit; and making plans to continue self-care after the study was completed [17]. The SMC component referred to the medication counselling provided by pharmacists to patients upon dispensing a prescription [17]. This study found that at the 3-month assessment, 6.7% of BMI-MTM patients reported continued misuse in comparison to 43.8% of SMC patients, and greater improvements in pain and depression were seen in the BMI-MTM patients [17]. The feasibility and acceptability of the BMI-MTM intervention was demonstrated by the high screening, consent, intervention completion, and follow-up rates in the study, along with the reported high level of patient satisfaction [17]. Due to the small sample size, the study noted that future research should build on this preliminary data within a fully powered clinical trial to potentially support broader application of the intervention [17]. Overall, this study lends preliminary support to the concept that a multicomponent intervention is beneficial to ensuring better pain management as well as reducing opioid-related risks.

A larger randomized controlled trial was recently conducted in Australia [18]. The Chronic Pain MedsCheck (CPMC) intervention was an in-pharmacy, patient-centered, multicomponent service that focused on reviewing patient’s medications and providing education and information to improve their self-management of chronic pain [18]. The CPMC trial had two arms; Group A pharmacies offered an initial consultation and a follow-up consultation three months later, and Group B pharmacies offered an initial consultation and two follow-up consultations at 6 weeks and 3 months after the initial consult [18]. This multicomponent intervention included a pharmacist continuing education component, a pharmacist-directed medication review, access to trial resources, and a patient education component [18]. A total of 550 pharmacies had at least one participant start the CPMC Trial and complete their initial consultation, with a total of 8239 participants completing the initial consult, and 4374 participants completing the follow-up(s) [18]. Overall, the CPMC intervention delivered by Group A and Group B pharmacies was effective and statistically significant in improving severity of pain, degree of pain interference, psychological distress, and pain self-efficacy scores [18]. There was no change in the average daily morphine equivalent dose in either group [18]. Most of the participants (81.7%) felt their overall knowledge and understanding of their chronic pain medication had improved as a result of the pharmacist intervention, and around a fifth reported noticing a definite improvement that has made a real and worthwhile difference [18]. Overall, Group B showed greater improvements in most of the participants’ health outcomes at three months compared to Group A [18]. It is important to note that the high dropout rate in this study may potentially increase the risk of bias, and the authors of the CPMC trial noted that a longer trial period would be required to sufficiently measure the long-term effect of the intervention on the average daily morphine amount [18].

Veettil et al. conducted a systematic review of pain management interventions involving pharmacists and the impact of these interventions on pain intensity [15]. They found that interventions are more likely to be effective in reducing pain intensity, especially in chronic pain patients, if they are multicomponent interventions, i.e., the intervention includes a medication review or any other pharmaceutical care service (e.g., telephone interviews, dosage adjustments, nonprescription drug recommendations, etc.) in addition to a patient educational component [15]. Although this review could not identify which components would be most effective and under which conditions as part of a multicomponent pharmacist intervention, it does highlight the need to incorporate multiple components in any pharmacist-led intervention strategy to manage patients with chronic pain as patient education alone may not be sufficient [15].

Management of Other Co-morbidities—One study identified that untreated depression, anxiety, and insomnia were often common care gaps identified by pharmacists in patients prescribed opioids [19]. Manzur et al. conducted a pilot study to evaluate care gaps in risk and harm reduction strategies for patients prescribed opioids and to describe the implementation of a pilot pain management program in community pharmacies [19]. Pharmacists involved in the pilot study conducted comprehensive patient assessments prior to their appointment with their primary care provider [19]. Patients were seen over a span of 1 to 2 visits with the pharmacist, with a total of 19 visits documented during the study period [19]. Pharmacists commonly identified unaddressed issues with mood (68%), and addressed these co-morbidities by making recommendations to prescribers to initiate adjuvant medications for concomitant conditions (84%), dose adjustment (58%), and laboratory tests (74%) and by recommending nonpharmacological therapies to improve pain, mood, and sleep [19].

Continuing Education—Studies have found that the inclusion of continuing education for pharmacists is important to increase their ability and confidence to provide pain and opioid management interventions [20,21,22,23]. Thakur et al. found that the levels of self-efficacy and confidence of pharmacists to provide pain and opioid management are significant barriers to pharmacists proactively engaging patients who have an opioid prescription and offering their expertise [20]. Thakur et al. also found that pharmacists recognize their role as opioid stewards but express low confidence, time and training as barriers, stating that there is a need for structured training and resources for pharmacists to improve confidence and participation in pain management interventions [20]. Low confidence can affect pharmacist participation in services such as counselling patients on opioid risks, dispensing naloxone, educating on opioid storage and disposal, utilizing prescription drug monitoring programs, offering opioid deprescribing, and providing resources for addiction treatment [20]. Similarly, Nielson et al. found that pharmacists who have lower confidence in identifying unmanaged pain in patients on opioids are likely to have lower engagement in interventions like screening tools [21]. They found that each additional decade of practice for pharmacists was associated with a 31% reduction in the number of times they undertook screening of patients using the author’s screening tool for opioid outcome monitoring [21].

As part of a pilot in Australia to test the implementation of software called Routine Opioid Outcome Monitoring (ROOM), Nielson et al. examined whether the training and support provided to pharmacists to deliver ROOM increased pharmacists’ clinical knowledge and confidence on opioid safety [22]. Pharmacist training included a 1 h (live or prerecorded) webinar about delivering the ROOM intervention, which included information on the three-item pain scale to measure pain outcomes by assessing pain intensity and interference; how to screen for opioid use disorder, depression, risky alcohol use, and opioid side effects; and relevant counselling points to be used in the event of a positive screen [22]. To assess pharmacist engagement with the training and to ensure pharmacists could apply the information after the training in practice, knowledge assessment questions were embedded throughout the webinar [22]. Additional professional development resources were also available to the pharmacist upon request for self-directed learning after the webinar [22]. The study found that following training and implementation of ROOM, pharmacists’ confidence in identifying and responding to most opioid-related problems, such as unmanaged pain, depression, and opioid dependence, significantly increased compared to the baseline, which highlights the importance of incorporating continuing education for pharmacists as part of the implementation of a pain management program.

### 3.2. Barriers for Implementation

Several barriers have been identified in the literature that impact community pharmacists’ abilities to consistently provide specific pain and opioid management interventions. Pharmacy Workflow—Concerns about pharmacy workflow or time availability have been cited as a potential barrier to the implementation of new interventions [20,23,24]. Frenzel et al. used the theory of planned behaviour in a mixed-methods study to determine what contributes to the unsuccessful implementation of opioid risk screening [23]. Seventeen pharmacists completed the survey and 35% of pharmacists reported that the workflow of the pharmacy does not allow for additional time spent for opioid risk screening [23]. Similarly, Fleming et al. conducted a qualitative study to determine the beliefs of pharmacists on their willingness to engage patients (i.e., provide interventional counselling) with suspected substance misuse [24]. Thirty-one community pharmacists participated, and the most prevalent barrier to engagement was the additional time required for counseling, which may have a negative impact on normal pharmacy workflow and other dispensing tasks [24].

Attitudes, Beliefs, and Stigma—Pharmacists’ stigma and beliefs or attitudes about opioids may prevent uptake of opioid stewardship interventions [24,25]. For example, in the study conducted by Fleming et al., pharmacists reported that in some instances, they may not implement opioid risk screening because they believe that patients do not understand nor appreciate the importance and benefit of gathering a comprehensive history to guide patient care [24]. Furthermore, Cid et al. conducted a scoping review of community pharmacy-based naloxone programs and specific program interventions, and found that stigma from both the pharmacist and patient perspectives exists [25]. For example, patients may not be willing to approach pharmacists to discuss issues such as the need for a naloxone kit due to feelings of judgement [25]. While pharmacists are less comfortable forming therapeutic relationships with patients who misuse opioids and do not proactively approach patients to offer overdose prevention education [25]. Werremeyer et al. conducted a survey study where they examined the degree to which pharmacists prefer social distance from patients with opioid misuse and OUD using a social distance scale (SDS) [26]. Of the 187 pharmacists who completed the survey, the mean SDS score was 16.32 (range 9–23), where higher scores represented greater preference for social distance [26]. More than 59% of pharmacists had a SDS score greater than 15, which demonstrated an overall lack of willingness to interact with and stigmatization towards patients who misuse opioids or who have OUD [26]. Studies have found that incorporating continuing education, and resources for pharmacists to decrease stigma and support effective communication are solutions for mitigating these barriers [27,28]. Werremeyer et al. conducted an additional study where they implemented a training program for pharmacists to reduce stigma towards people who misuse opioids and measured social distance scores and negative attitudes through a survey pre- and post-program (immediate and after 12 months) [27]. This training program resulted in significantly lower social distance scores immediately post-program when compared to the baseline score (14.75 vs. 16.57, *p* = 0.000). The 12-months mean SDS score was also significantly lower than the baseline SDS score (15.32 vs. 16.57, *p* = 0.017) [27]. Significant changes in negative attitudes from baseline to post-survey and from baseline to 12 months were also demonstrated [27]. Similarly, Eukel et al. investigated the changes in pharmacists’ perceptions following a training program on opioid misuse and overdose prevention and found that the training improved knowledge and stigma [28]. Statistically significant changes to perceptions were noted after the training program in the following areas: opioid addiction being outside the control of the patient, the role of family history in prescription drug abuse, the value of screening and counselling to support patients at risk of prescription opioid abuse, and the importance of seeing things from the patient’s perspective [27].

Remuneration—Studies involving additional clinical services in pharmacies often report that pharmacists are likely to continue to provide the service if there is compensation for it [21]. In the study by Nielson et al., pharmacists were compensated AUD $20 for completing baseline and follow-up surveys for the research study, AUD $40 for completing the training webinar, and AUD $20 for each ROOM intervention completed with a patient [21]. Just under half of the participating pharmacists (44%) indicated that they were very likely to continue to provide the intervention as long as they continued to have access to the software at no charge and were provided a professional service fee [21]. In comparison, only one pharmacist responded that they were very likely to continue to provide the service if no professional service fee was provided [21]. Similarly, Alenezi et al. conducted a qualitative study of community pharmacists’ roles, barriers, and behavioural determinants related to involvement in optimizing opioid therapy for chronic pain and found that systemic constraints, such as a lack of funding for the professional service was a major barrier, and in order for further uptake, the intervention needed to be appropriately funded [29].

### 3.3. Facilitators for Implementation

The literature search identified one facilitator as the pharmacist’s expanded scope of practice in Canada [30]. Expanded Scope—In response to the COVID-19 pandemic, in March 2020, to facilitate continuity of care for vulnerable patients on opioids, Health Canada issued a subsection 56(1) class exemption from the *Controlled Drugs and Substances Act* (CDSA), which permitted pharmacists to extend and renew prescriptions, transfer prescriptions to another pharmacist, and receive verbal orders from prescribers for controlled substances, in addition to permitting pharmacy employees to deliver prescriptions for controlled substances to patients [30,31]. As these exemptions are subject to the laws and regulations of the province or territory where the pharmacist is entitled to practice, jurisdictions underwent regulatory amendments as required to incorporate some or all of these activities into the scope of practice of their pharmacists [32,33]. Some jurisdictions have also enabled pharmacists to adapt prescriptions for controlled substances, which was already permitted under the CDSA [30,33]. Bishop et al. conducted a qualitative study to explore the perceptions of Canadian pharmacists about the barriers and facilitators of providing opioid stewardship activities when considering this expanded scope of practice [30]. Twenty pharmacists from all provinces and from urban and rural practices were interviewed and reported that the Health Canada CDSA exemptions facilitated their ability to provide opioid stewardship and positively impacted patient care by providing continuity of and timely access to care [30].

## 4. Discussion

This review demonstrated that when designing an effective pharmacist intervention for pain and opioid management, consideration should be made to include multiple components in the pharmacist intervention as opposed to only patient education [15,17,18]. In addition to the intervention being multicomponent, one of the components should target treatment of psychopathology, which is often a comorbidity found amongst chronic pain patients and are often predictors of opioid abuse, misuse and overdose [19]. The intervention should also include a continuing education component, as this review has showcased that the inclusion of continuing education for pharmacists is important to increase their ability and confidence to provide the intervention [20,21]. Continuing education as part of the implementation of a pain management program also reinforces and supplements the knowledge and training that pharmacists already have [21]. In addition to improving self-efficacy and confidence, continuing education also decreases the negative attitudes, beliefs and stigma pharmacists may have towards patients who consume opioids and/or have OUD, and consequently lead to changes in their behaviour and an improvement in patient outcomes [27,28]. Although multicomponent interventions have shown to have benefits in small-scale pain management interventions, one downfall is that they may be more complex and therefore harder to implement on a large scale. Future research should explore whether a multicomponent vs. a targeted intervention is most effective for large-scale implementation to encourage further uptake and positive outcomes.

In addition to these components, it is important for future researchers developing an opioid and pain management intervention to include appropriate renumeration for the pharmacists participating in the intervention [21,29]. The remuneration model for community pharmacies has transitioned over recent years from one primarily focused on dispensing services to include an increasing number of funded clinical services [34]. Traditionally, pharmacies are renumerated through their Usual and Customary fee for the dispensing of prescriptions to ensure technical accuracy and therapeutical appropriateness, and this includes providing basic counselling and patient education as part of that service [35]. Renumeration for additional clinical services are not included within the traditional dispensing model, and if no alternative funding is available, this may result in a limited capacity for pharmacists to deliver these services. As more studies continue to report the clinical benefits of pharmacist cognitive services, and pharmacy revenues from dispensing continue to decrease due to generic drug price reductions, pharmacists report that advocating for appropriate payment for patient care services is needed [35,36]. Based on the previous uptake of pharmacy programs in Canada, pharmacists are generally more willing to implement additional services when there is an associated professional fee for the additional time it takes to provide the service [35,37]. Therefore, funding to support professional service fees would likely result in greater uptake with more pharmacists choosing to offer additional interventions.

Pharmacy workflow challenges were also discussed as a barrier to intervention implementation. These challenges can further be addressed by encouraging pharmacists to use an appointment-based model where patients schedule an appointment with the pharmacy to allow for better organization of tasks [38]. In addition, challenges to workflow can be addressed by optimizing pharmacy staffing, leveraging the expanded scope of pharmacy technicians, and if enabled and compensated through remuneration for the service, hiring additional staff. The expansion in scope of practice for Canadian pharmacists via the Health Canada CDSA exemption is a major facilitator for pharmacists to further engage in opioid stewardship by removing barriers such as requiring a new prescription authorization from a prescriber for dosage adjustments to undertake opioid deprescribing [33]. However, the exemption is time-limited and is set to expire on September 30, 2026 [32]. Given the benefits realized from this exemption, careful consideration should be given to the continuation of the CDSA exemption permanently to facilitate future interventions involving pain management, and support better access to timely healthcare for patients.

### 4.1. Future Studies

In completing the narrative review, there was only a small number of studies conducted from the Canadian perspective, therefore, future research should focus on implementing Canadian pharmacy-specific interventions to combat the opioid crisis, as the scope of practice for pharmacists differs between countries and research involving Canadian pharmacy professionals would be useful for developing future policies and programs in Canada. This review highlighted interventions from other jurisdictions and suggested that there is a significant impact that pharmacists could have on pain management and the opioid crisis. Future studies should include measures for feasibility and cost, as expanded pharmacy services could translate to cost savings for the government while also considering the cost to implement and sustain a new intervention. An analysis from the Canadian Pharmacists Association on other pharmacy services such as smoking cessation, advanced medication review for heart disease, and pneumococcal vaccination revealed that Canada-wide implementation of these services could yield cost savings of CAD 2.5 billion to CAD 25.7 billion over the next 20 years depending on program uptake [33]. Research has also showcased that community distribution of naloxone, which is publicly funded and is available through community pharmacies in Canada, not only saves lives but is also a cost-effective strategy for combating the opioid crisis [39,40]. However, an appropriately powered cost–benefit analysis has yet to be conducted on pharmacist pain management interventions, and future studies are required to demonstrate costs associated with implementation and any cost savings for the healthcare system [40].

### 4.2. Limitations

There are several limitations to this review. First, this literature review was conducted as a narrative review, which is typically meant to be a comprehensive review of available knowledge on a topic, but runs the risk of reviewer bias due to a lack of a systematic search strategy which is typically found in systematic or scoping reviews [41]. The authors did not have sufficient time or resources available to conduct a systematic review due to funder-mandated deadlines; therefore, a narrative review was conducted. Second, pharmacist-led pain management interventions, like the ones found in this review, are often pilot studies and, therefore, their long-term effect is not known. Future research involving such interventions should include measuring their long-term impact.

## 5. Conclusions

While all healthcare professionals in a patient’s circle of care can contribute as opioid stewards, this review highlights specifically the role that pharmacists, as medication experts, can play in opioid stewardship by improving, monitoring, and evaluating the use of prescription opioids. Since prescription opioids are one of the contributors to the growing opioid crisis in Canada, a potential solution involves enabling pharmacists to provide more in-depth pain management services beyond what is provided when dispensing prescriptions. This could be done by developing a community-based pharmacist-led intervention for managing chronic pain in patients who are taking opioids. Pharmacists being conveniently located in all communities and often available for extended hours, can provide accessible and timely care for patients. They also have the ability to provide more frequent follow-ups by virtue of being seen more often by patients, which may further improve the management of pain and reduce the risk of opioid-related harms, such as misuse and overdose, as well as the progression to opioid use disorder. Although the benefits of pharmacist interventions have been documented in other jurisdictions, there is a need to investigate the impact of a community pharmacist-led intervention in the context of the Canadian landscape. The establishment of a multicomponent intervention conducted in community pharmacies that can be scaled accordingly and integrated within the healthcare system may not only improve the care provided to patients, and consequently result in better patient health outcomes, but can also increase capacity in the system through efficient use of healthcare resources, for example by reducing the demand on primary care providers to provide patient’s with frequent follow-up for pain management.

## Data Availability

No new data were created or analyzed in this study. Data sharing is not applicable to this article.

## Appendix A

**Table A1:** Selected articles describing community pharmacist-led pain management programs.

Study Author	Associated Theme	Type of Study	Objective	Pharmacist Intervention	Results
Cochran et al. [17]	Multicomponent Interventions	Randomized Controlled Trial	To test the feasibility and acceptability of Brief Motivational Intervention-Medication Therapy Management intervention (BMI-MTM) with Standard Medication Counselling (SMC) compared to SMC alone.	BMI-MTM is comprised of medication therapy management (MTM), brief motivational interviewing (BMI), patient navigation, naloxone training and referral.	Thirty-two recipients were included in the trial. BMI-MTM demonstrated feasibility through all intervention recipients completing the study. BMI-MTM recipients indicated ≥4.2 out of five levels of satisfaction with the pharmacist-led session, and 92.4% were satisfied with the patient navigation sessions. When compared to SMC at 3 months, BMI-MTM recipients reported greater improvements in opioid misuse.
Medical Services Advisory Committee [18]	Multicomponent Interventions	Randomized Controlled Trial	To test the efficacy of the Chronic Pain MedsCheck (CPMC) intervention in preventing incorrect use and/or overuse of pain medication, increasing participant’s health literacy, improving their ability to self-manage their chronic pain and improving their overall quality of life. Additionally, to determine the level of acceptance and satisfaction with, by pharmacists, participants and referred providers, and the cost-effectiveness/utility of the CPMC intervention.	The CPMC intervention was multicomponent in that it included a pharmacist continuing education component, a pharmacist-directed medication review, access to trial resources, and a patient education component. Group A pharmacies offered an initial consultation and a follow-up consultation three months later, while Group B pharmacies offered the initial consultation and two follow-up consultations 6 weeks and 3 months after the initial consult.	A total of 550 pharmacies participated, with 8239 patients completing the initial consult, and 4374 patients completing the follow-up(s). The CPMC trial delivered by Group A and Group B pharmacies were effective and statistically significant in improving severity of pain, degree of pain interference, psychological distress, and pain self-efficacy scores. Overall, Group B showed greater improvements in most of the participants’ health outcomes at three months compared to Group A. Group A participants improved their average self-management and health literacy total scores from initial to follow-up, and both increases were statistically significant. Group B participants also had a statistically significantly higher average self-management score at follow-up compared to initial but the increase in their average health literacy total score was not statistically significant. Both groups demonstrated statistically significant improvements in overall quality of life for patients. Most of the participants (81.7%) felt their overall knowledge and understanding of their chronic pain medication had improved as a result of the intervention. Pharmacists’ perceived ease of use of the intervention was mixed. ‘Following the intervention protocol’ and ‘using the mini-ePPOC tool’ were rated to be the easiest tools to use, and ‘developing an action plan’ was rated as being harder to perform. Incremental cost-effectiveness ratios (ICER) showed that Groups A and B are dominant to treatment as usual. Group B had a cost-saving ICER of CAD $2578.43 per unit of morphine lost. As there are no published ‘willingness to pay’ thresholds for the studies’ outcomes, the authors report that it is difficult to determine if these cost savings are acceptable.
Veettil et al. [15]	Multicomponent Interventions	Systematic Review and Meta-analysis	Summarize the effects of pharmacist interventions on pain intensity over time in individuals with pain of any etiology	N/A	Twelve randomized controlled trials including 1710 participants were included. A pooled estimate of the 12 studies demonstrated a statistically significant reduction in pain intensity compared with control. Interventions were more effective when they included a combination of services such as educational interventions, medication review, and pharmaceutical care services rather than educational interventions alone. High-quality randomized controlled trials are needed to confirm the clinical significance of these findings before advocating for widespread implementation in clinical practice.
Manzur et al. [19]	Management of Other Comorbidities	Pilot study	To evaluate care gaps in risk- and harm-reduction strategies for patients prescribed opioids and to describe the implementation of a community pharmacy-based, pilot pain-management program.	Patients were seen in the pharmacy before their appointment with the referring provider. Pharmacists conducted a comprehensive patient assessment with recommendations for provider implementation. The assessment included a detailed medication history; risk assessment using the Opioid Risk Tool; monitoring of state Prescription Drug Monitoring Program data; basic mood assessment with or without administration of the Patient Health Questionnaire-9; pain score assessment using a numeric pain-intensity scale; and assessment of pain, enjoyment of life, and general activity using the Pain, Enjoyment, General Activity (PEG) screening tool.	Patients were seen over a span of 1 to 2 visits; a total of 19 visits were documented. Pharmacists identified unaddressed issues with mood (68%). Recommendations made to the providers included additional therapy (84%), dose adjustments (58%), and laboratory tests (74%). Naloxone was provided (58%), and education on naloxone use was provided at every visit. Untreated depression, anxiety, and insomnia were the most common problems identified by pharmacists. Pharmacists implemented and documented risk-reduction strategies and co-prescribed naloxone more frequently compared with clinic providers. The program enhanced the pharmacists’ ability to make safe and clinically appropriate decisions regarding filling opioid prescriptions.
Nielson et al. [22]	Continuing Education	Pilot study	To test the implementation of software-facilitated Routine Opioid Outcome Monitoring (ROOM) tool.	The ROOM tool included information on the three-item pain scale to measure pain outcomes by assessing pain intensity and interference; how to screen for opioid use disorder, depression, risky alcohol use, and opioid side effects; as well as relevant counselling points.	Sixty-four pharmacists from 23 pharmacies were recruited and trained to conduct ROOM. Twenty pharmacies (87%) implemented ROOM. Pharmacists completed ROOM with 152 patients in total. Forty-four pharmacists provided baseline and follow-up data which demonstrated significant improvements in confidence identifying and responding to unmanaged pain, depression and opioid dependence. Despite increases, low to moderate confidence for these domains was reported at follow-up. Responses from pharmacists and patients indicated that ROOM is feasible and acceptable, though more extensive pharmacist training with the opportunity to practice skills may assist in developing confidence and skills.

**Table A2:** Selected articles describing factors to consider when implementing an opioid or pain management intervention.

Study Author	Associated Theme	Study Type	Objective	Results
Thakur et al. [20]	Continuing Education, Pharmacy Workflow	Commentary	To describe current and potential roles for pharmacists to combat the United States opioid crisis and identify key factors affecting service provision.	Pharmacists recognize their roles as counselling patients on opioid risks, dispensing naloxone, educating on opioid storage and disposal, using prescription drug monitoring programs, offering opioid deprescribing, and providing resources for opioid use disorder treatment. Pharmacists express low confidence, time, and training as barriers to service provision. There is a need for structured training, resources, and organizational support for pharmacists to improve confidence and participation in such services.
Nielson et al. [21]	Continuing Education, Renumeration	Cross-sectional study	To examine pharmacist characteristics associated with implementation of the Routine Opioid Outcome Monitoring Tool (ROOM)	Fewer years of practice was associated with a greater number of screenings conducted. Each additional decade of practice was associated with a 31% reduction in the number of screenings completed by pharmacists. Further analysis revealed that each additional decade of practicing was associated with lower knowledge of naloxone and lower confidence in identifying unmanaged pain and were all independently associated with reduced engagement in screening. About half of participating pharmacists (44%) indicated that they were very likely to continue to provide the intervention as long as they continued to have access to the software at no charge and were provided a professional service fee. Only one pharmacist responded that they were very likely to continue to provide the service if no professional service fee was provided.
Frenzel et al. [23]	Pharmacy Workflow; Attitudes, Beliefs and Stigma	Mixed Methods	To use the theory of planned behavior to determine what attitudes and beliefs contribute to the unsuccessful implementation of opioid risk screening.	Seventeen pharmacists completed the survey. Pharmacists indicated positive attitudes toward reducing negative opioid outcomes for patients using opioid medications. The highest proportion of negative responses was observed in the perceived behavioral control construct which included difficulty in offering the screening and unsuccessful integration of past interventions.
Fleming et al. [24]	Pharmacy Workflow	Qualitative Study	To elicit modal salient beliefs of community pharmacists regarding their willingness to engage patients (i.e., provide interventional counseling) with suspected controlled substance misuse	Thirty-one pharmacists participated. The most prevalent belief was the disadvantage associated with patient confrontations. Pharmacists also believed that engaging patients may cause loss of customers/business but may help patients receive appropriate counseling. Pharmacists identified regulatory agencies (e.g., pharmacy boards, law enforcement) and family/friends of patients as groups of individuals who influence their willingness to refer. Time required for counseling was found to be the most cited control belief or barrier.
Cid et al. [25]	Attitudes, Beliefs and Stigma	Scoping Review	To summarize the literature on community pharmacy-based naloxone programs, including specific program interventions as well as facilitators and barriers for naloxone programs, and knowledge gaps.	The top three barriers identified were: cost/coverage of naloxone, stigma, and education/training for pharmacists. Naloxone program interventions included screening tools, checklists, pocket cards, patient brochures, and utilizing the pharmacy management system to flag eligible patients. Patient knowledge gaps included naloxone misinformation and lack of awareness, while pharmacists demonstrated administrative, clinical, and counselling knowledge gaps.
Werremeyer et al. [26]	Attitudes, Beliefs and Stigma	Survey	To examine the degree to which pharmacists prefer social distance from patients with opioid misuse and opioid use disorder (OUD) using a Social Distance Scale (SDS).	Mean SDS total score was 16.32 (range 9–23), indicating overall lack of willingness to interact with the vignette patient. Females had a higher mean SDS score vs. male pharmacists (16.58 vs. 15.36, respectively; *p* = 0.023). Pharmacists with >10 years of experience, without personal experience with a substance use disorder, or who strongly agreed that patients with OUD require excessive time and effort, and those who agreed that some people lack self-discipline to use prescription pain medication without becoming addicted had significantly higher SDS scores.
Werremeyer et al. [27]	Continuing Education, Attitudes, Beliefs and Stigma	Cohort Study	To measure changes in social distance scale (SDS) total score from baseline to post-survey and from baseline to 12 months, as well as change in SDS question scores and change in negative attitudes.	The mean total SDS score was significantly lower in the immediate post-training survey than the pre-training mean (14.75 vs. 16.57, *p* = 0.000). The 12 months mean total SDS score was also significantly lower than the pre-training mean (15.32 vs. 16.57, *p* = 0.017). Significant changes in negative attitudes from baseline to post-survey and from baseline to 12 months were seen.
Eukel et al. [28]	Continuing Education, Attitudes, Beliefs and Stigma	Cohort Study	To describe the results of education-related training to promote behavioral change by altering pharmacists’ perceptions toward opioid misuse.	Five items showed a statistically significant (*p* < 0.05) change in perceptions after the training. Significant changes were reported for opioid addiction being outside the control of the affected person, the role of family history in substance misuse, the value of counseling to support patients at risk of opioid misuse, the value of screening tools, and the importance of viewing things from the patient’s perspective.
Alenezi et al. [29]	Renumeration	Qualitative study	To determine community pharmacists’ roles, barriers, and behavioural determinants related to involvement in optimizing opioid therapy for chronic pain.	Pharmacists demonstrated a desire to contribute to opioid therapy optimization. However, they described barriers to optimization as a lack of knowledge, skills and training, inadequate time and resources, systemic constraints, and other barriers, including relationships with doctors and patients.
Bishop et al. [30]	Expanded Scope	Qualitative Study	To explore the perceptions of Canadian pharmacists about the barriers and facilitators of providing opioid stewardship activities in pharmacy practice, considering the subsection 56(1) class exemption under Health Canada’s Controlled Drugs and Substances Act (CDSA).	Twenty pharmacists from community and primary healthcare teams, from all provinces and from urban and rural practices were interviewed. The following themes included: (1) optimization of opioid-related patient care, (2) jurisdictional impact and (3) awareness and education. Barriers and facilitators for opioid stewardship activities were identified. The exemptions facilitated pharmacists’ ability to provide opioid stewardship and positively affected patient care by providing continuity of and timely access to care.
